# A rare case of eccrine spiradenoma—treatment and management

**DOI:** 10.1007/s00238-015-1103-4

**Published:** 2015-06-17

**Authors:** Subha Dhua, D. R. Sekhar

**Affiliations:** Department of Plastic and Reconstructive Surgery, Vydehi Institute of Medical Sciences and Research Centre, Bangalore, Karnataka India

**Keywords:** Brooke–Spiegler syndrome, Z-plasty, *CYLDI* gene, Eccrine unit, Multiple familial trichoepithelioma (MFT)

## Abstract

A young male patient presented with multiple swellings on his chest and the nape of his neck. Physical examination revealed multiple small papulonodular swellings measuring 0.5 × 0.5 cm to 2 × 2 cm, that were soft without discharge with no surrounding skin changes or induration. Skin biopsy samples were diagnosed as benign adnexal neoplasm consistent with eccrine spiradenoma, trichoepithelioma, and cylindroma, i.e., Brooke–Spiegler syndrome. Having confirmed this to be a case of eccrine spiradenoma, surgical excision was performed and the raw area was covered with a split thickness skin graft taken from the right thigh and sutured over the raw area. The sternal lesion was circumferentially excised and the wound was primarily closed by Z-plasty. Surgical excision is considered the gold standard for the treatment of these cases, with low rates of recurrence. Around 50 such cases have been reported in the literature to date. Although eccrine spiradenomas are usually solitary and small, the findings in our case underscore the fact that a variety of presentations are possible. With strict clinical suspicion and histological criteria, the correct diagnosis can be achieved, especially when combined with pertinent clinical information and laboratory studies.

Level of Evidence: Level V, therapeutic study.

## Introduction

Eccrine spiradenoma is an adnexal neoplasm that continues to be designated as a tumor with eccrine differentiation, however, it is currently considered an apocrine process. The disease usually appears as solitary gray, purple, pink, red, or blue nodules on the upper half of the body. These multiple, potentially painful spiradenomas were reported for the first time by Kerstein et al [1].

Eccrine spiradenomas are usually benign and mostly occur in patients aged 15–35 years [2]. To date, about 15 cases of linear/zosteriform/nevoid/blaschkoid multiple spiradenomas have been reported [3, 4]. However, 50 cases of malignant spiradenoma exist in the literature, and Dabska et al. [5] were the first to describe malignant spiradenoma in 1972.

While eccrine spiradenomas are indeed rare worldwide, the malignant variant is rarer still.

Eccrine spiradenomas can be painful, but the rate of malignant transformation is very low. Such transformation to malignancy has been reported to develop spontaneously with a metastasis rate of 50 %, which can cause death.

In the reported literature, no sex predilection exists for spiradenomas or malignant spiradenomas. Although the exact etiology of these lesions is unknown, spiradenomas appear to be caused by a defective tumor suppressor gene; in Brooke–Spiegler syndrome (BSS), there is usually a defect in the *CYLDI* gene located on chromosome 16 at position 12.1 [4].

## Case report

A 25-year-old patient presented with a history of insidious onset of multiple swellings over the chest and the nape of his neck since 5 years of age, which gradually progressing and was associated with an itching and burning sensation. There were no other co-morbid illnesses or significant history.

Physical examination revealed multiple small papulonodular swellings measuring 0.5 × 0.5 cm to 2 × 2 cm, which had a soft consistency with no discharge and no surrounding skin changes or induration.

**Fig. 1:**
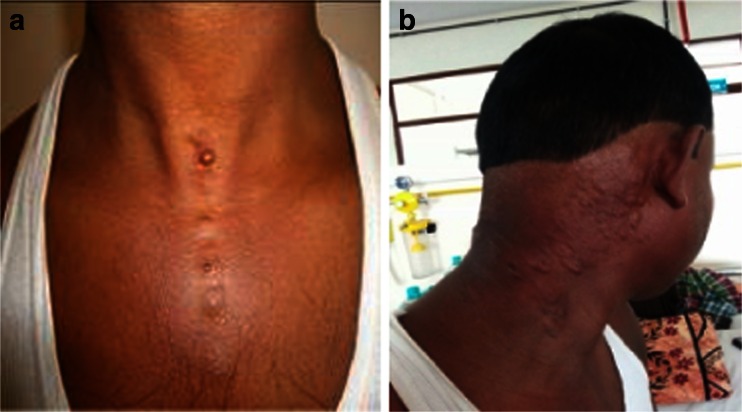
**a**, **b** Skin showing multiple ill-defined small nodules

Lesions were confined to the skin with no deeper structure involvement (Fig. 1a, b).

The raised nodules were tender, firm, fixed to the skin, and ranged from 0.5 to 6 cm in diameter. For histological analysis, surgical excisions measuring 24 × 11 cm and 6 × 13 cm were sent for examination.

**Fig. 2:**
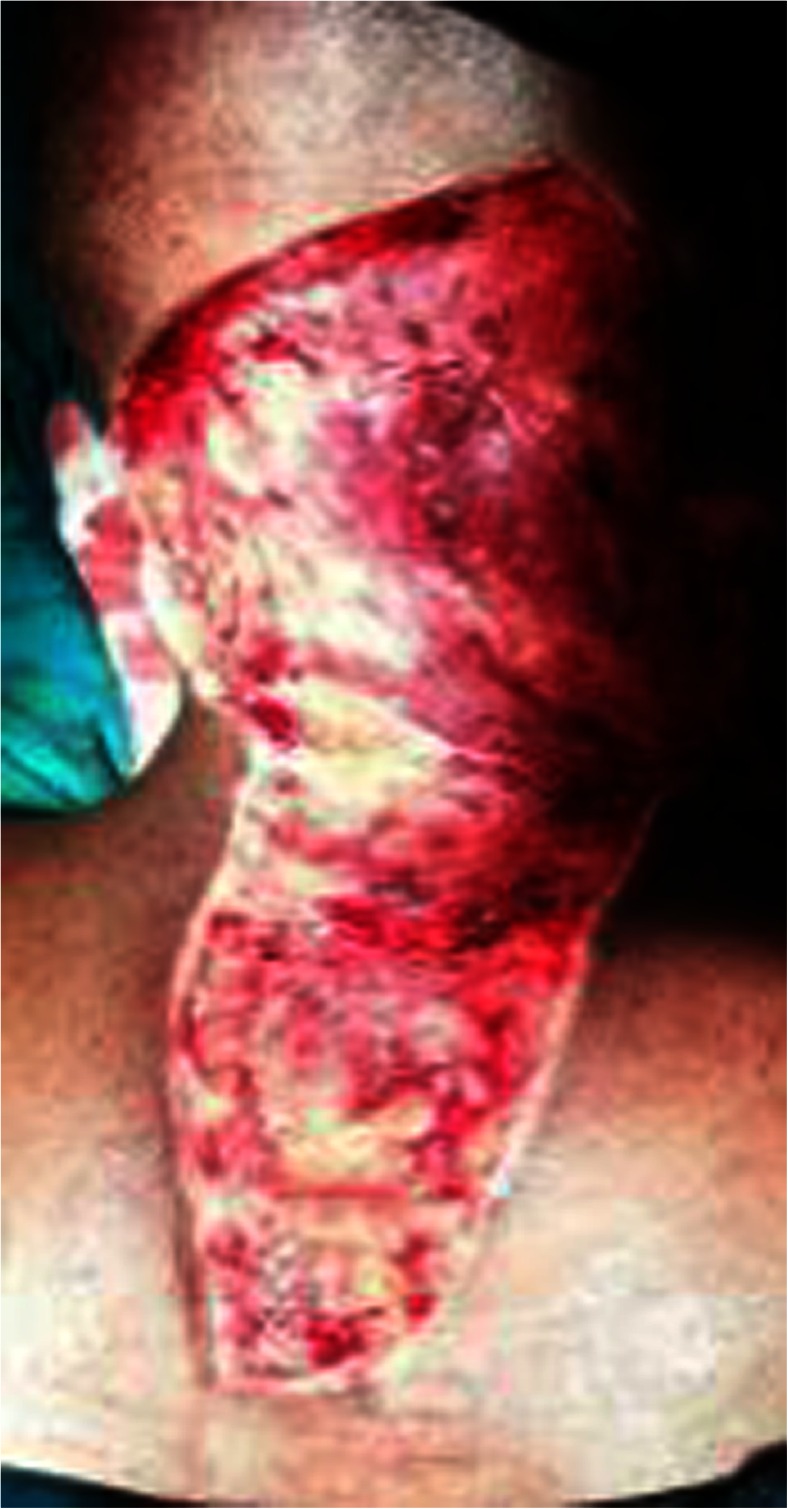
Surgical excision of the lesion extending between the Occipital Region and in the Nape of Neck measuring 24 cm × 11 cm

**Fig. 3:**
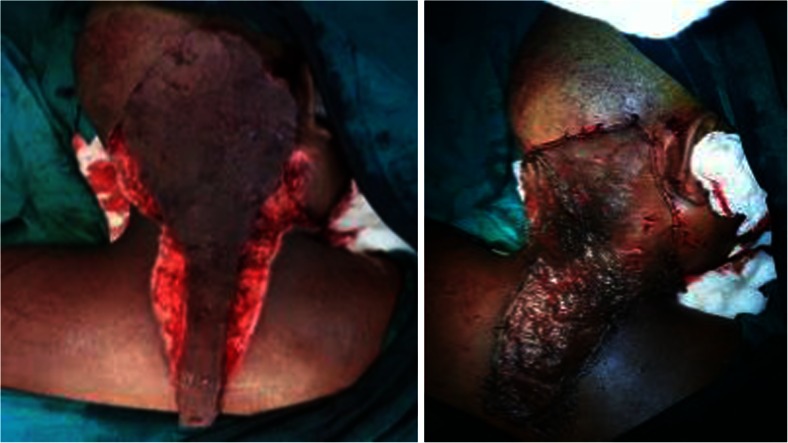
Excised area covered by split thickness skin graft

**Fig. 4:**
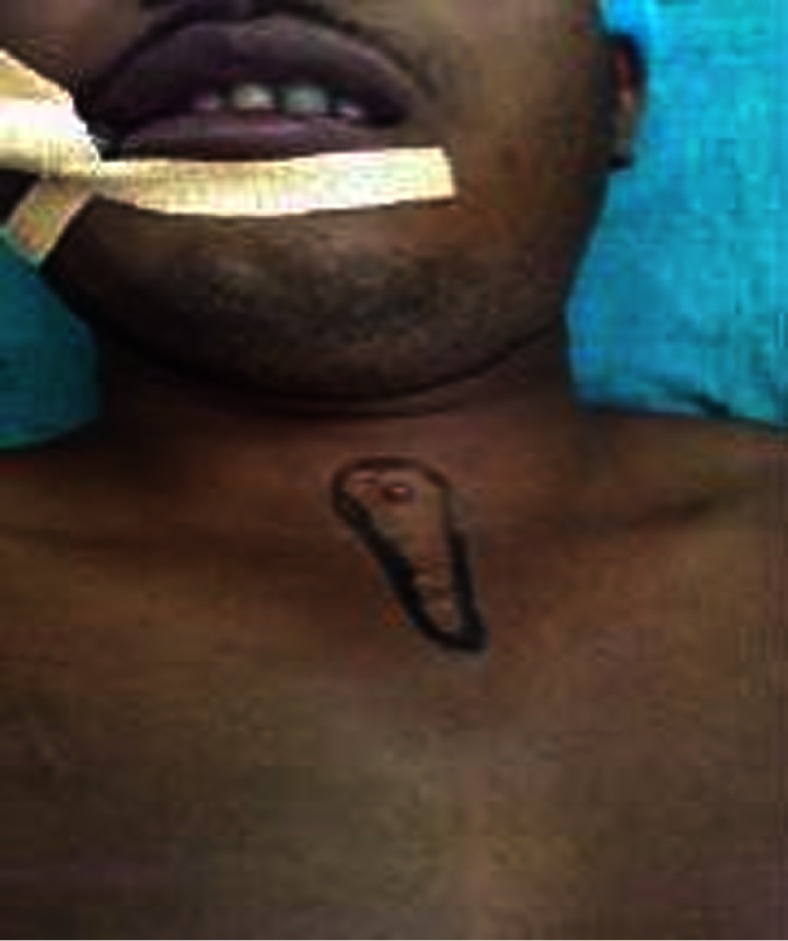
Markings at Pre-tracheal area for conducting surgical excision

**Fig. 5:**
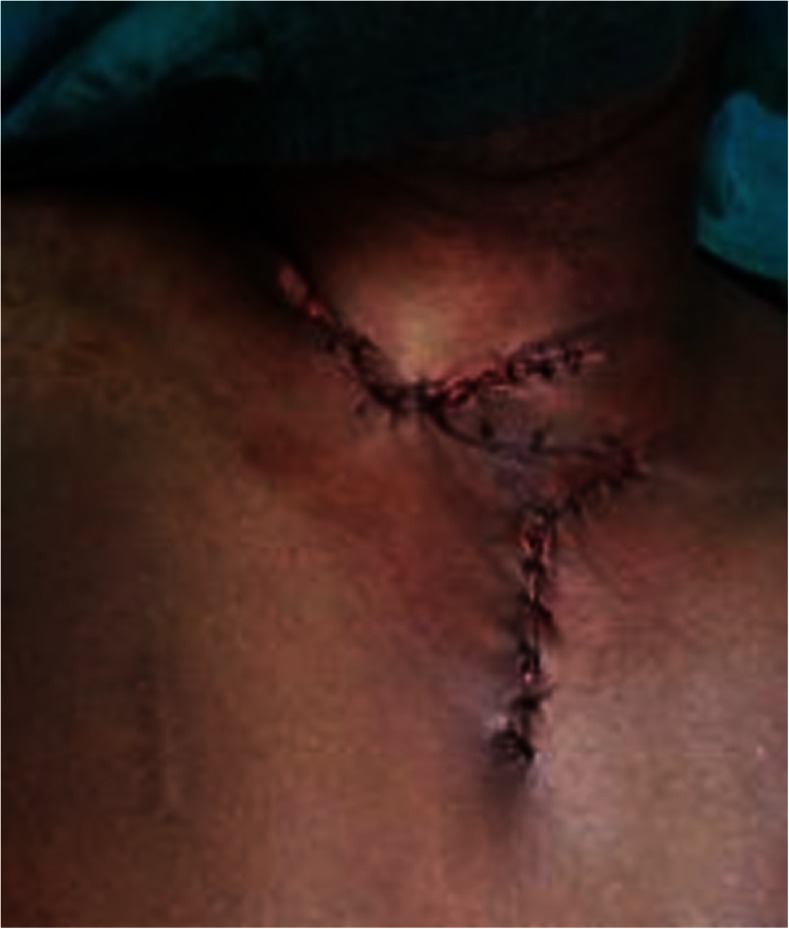
Wound at the pre-tracheal and pre-sternal area closed primarily by Z-plasty

The patient was turned to the prone position, and the lesions were circumferentially excised from the occipital region to the nape of the neck to the back after marking for surgical excision (Fig. 2). The raw area was covered with a split thickness skin graft taken from the right thigh and sutured over the raw area (Fig. 3). The pre-tracheal and pre-sternal lesion was circumferentially excised, and the wound closed primarily by Z-plasty (Figs. 4 and 5).

The two skin biopsies were independently diagnosed as benign adnexal neoplasm consistent with eccrine spiradenoma, trichoepithelioma, and cylindroma, i.e., BSS [7].

**Fig. 6:**
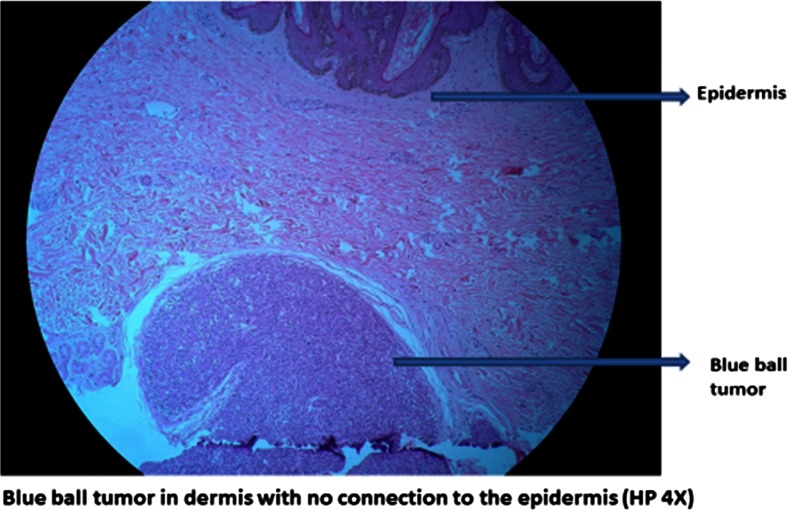
*Blue ball tumor* in dermis with no connection to the epidermis (HP ×4)

**Fig. 7:**
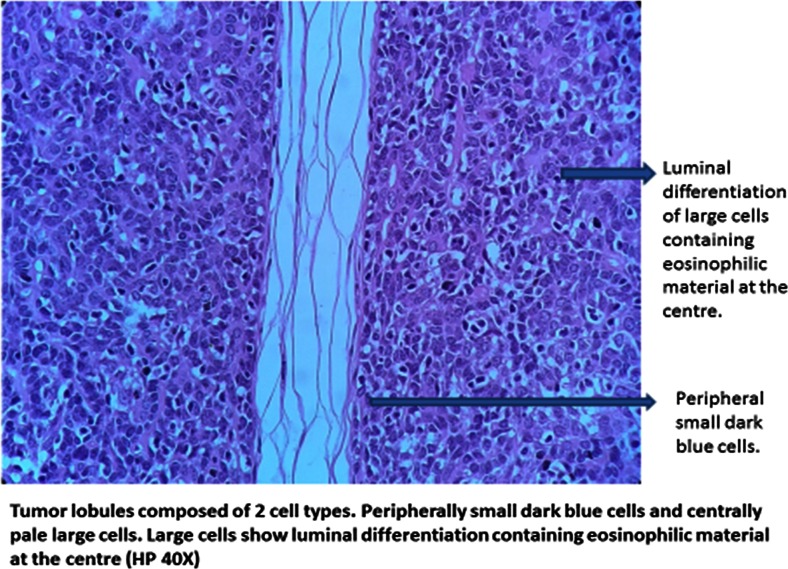
Tumor lobules composed of two cell types. Peripherally small dark blue cells and centrally pale large cells. Large cells show luminal differentiation containing eosinophilic material at the center (HP ×40)

Multiple sections from both the tissue masses show a lesion centered in the deep dermis. The epidermis appeared unremarkable, and the multiple tumor nodules were of varying sizes situated in the dermis and not connected to the epidermis. They were oval or spherical with tightly packed cells arranged in diffuse, alveolar, or pseudo-rosette formations. The tumor nodules have two populations of cells: a large cell type with a vesicular nucleus and prominent nucleolus with scanty cytoplasm, and a small cell type with a condensed dark nucleus that is located at the periphery of the nodules. There is evidence of eosinophilic, basement membrane-like material around the lobules. However, the majority of the tumor lobules are surrounded by well-defined fibrous capsules. Some nodules show cystic changes, and mild lymphocytic infiltrate was noted throughout the tumor surrounding the nodules. There was no evidence of horn cysts and no evidence of hyaline material around the tumor nodules (Figs. 6 and 7).

These are characteristic benign neoplasms that show a substantial degree of differentiation towards eccrine. They consist of two cell types: peripheral small dark cells and central large pale cells. These cells are arranged in well-defined lobules (blue balls in the dermis) with no connection to the overlying epidermis. These cells sometimes form primitive eccrine coils and luminal structures. The histopathologic hallmark of eccrine spiradenoma is well demarcated lobules composed of two types of cells [6].

In the present case, special care was taken to ensure proper split thickness skin graft take. The excised area provided a well-vascularized scar-free bed on which the split thickness skin graft taken from the right thigh was sutured over the raw area. Absence of infection was ensured at the recipient site by preparing the wound bed, using quality sutures and applying regular dressings. Also, care was taken to ensure the absence of shear forces to prevent separation of the graft from the bed for graft survival. Meticulous hemostasis during operation was ensured to prevent hematoma formulation, and the operation steps were executed to give the bed a prolonged period for the normal hemostatic process to take effect. As the graft take was satisfactory, the wound healing time was normal and uneventful. The patient was followed up over a period of 6 months, and no recurrence was clinically observed. The patient was instructed to report for a follow-up after a period of another 6 months to ensure that there was no early recurrence.

## Discussion

Eccrine spiradenoma is a benign adnexal tumor that appears as a small and bluish nodule that is typically tender on palpation. In our case, multiple tumors were present on the exterior and the interior surface of the upper body. Random multiple tumors have been reported on the chest, upper extremities of the forehead, and scalp [7]. Moreover, linear distributions have also been reported [8, 9]. A case of multiple “nevoid” spiradenomas with lesions constrained to the right half of the patient’s body from the face to the lower limb has been reported by Noto et al. [10]. It is noteworthy that these lesions and the ones in the present study followed Blaschko lines because of the involvement of multiple dermatomes.

The literature shows that the onset of nodules may occur at birth. However, they are commonly seen in the second to third decade of life, are more common in females, and occur on the neck and the back. Affected individuals are also at a higher risk of developing benign or malignant tumors of the salivary glands. BSS is an autosomal dominant condition, caused by mutations in the *CYLD* gene; genetic studies have identified a single gene, *CYLD1*, on 16q12-q13 as being altered in BSS. The penetrance of this gene has been estimated to be between 60 and 100 %. However, mutations in the *CYLD1* gene are also found in familial cylindromatosis and familial trichoepithelioma. Therefore, histopathology plays an important role in distinguishing between BSS, FC, and MFT.

The various treatment modalities include excision, dermabrasion, electrodessication, cryotherapy, and radiotherapy or applying argon and CO_2_ lasers. It has also been shown that the administration of aspirin and its derivatives can result in the rapid formation of new lesions.

Although eccrine spiradenomas are usually solitary and small, the findings in our case underscore the fact that a variety of presentations are possible. This case also shows that eccrine spiradenoma is another dermatologic condition that can be distributed along Blaschko lines. The etiology of this condition, which is undoubtedly genetic, needs further investigation. However, the diagnosis is of paramount importance because of potential malignant transformation particularly in the case of multiple or symptomatic lesions. With strict clinical suspicion and histological criteria, the correct diagnosis can be achieved, especially when combined with pertinent clinical information and laboratory studies. Treatments for spiradenoma have not yet been clearly established, but surgical excision is still the gold standard with low rates of recurrence.
